# Heel lance in newborn during breastfeeding: an evaluation of analgesic effect of this procedure

**DOI:** 10.1186/1824-7288-34-3

**Published:** 2008-11-18

**Authors:** Elena Uga, Manuela Candriella, Antonella Perino, Viviana Alloni, Giuseppina Angilella, Michela Trada, Anna Maria Ziliotto, Maura Barbara Rossi, Danila Tozzini, Clelia Tripaldi, Michela Vaglio, Luigina Grossi, Michaela Allen, Sandro Provera

**Affiliations:** 1 Department of Paediatrics, S. Andrea Hospital-ASL VC, Vercelli, Italy; 2 Department of Paediatrics, Hospital of Putignano-ASL BA, Bari, Italy; 3 General Practitioner, ASL Pavia, Pavia, Italy

## Abstract

**Objectives:**

The reduction of pain due to routine invasive procedures (capillary heel stick blood sampling for neonatal metabolic screening) in the newborn is an important objective for the so-called "Hospital with no pain". Practices such as skin to skin contact, or breastfeeding, in healthy newborn, may represent an alternative to the use of analgesic drugs. The aim of our work is to evaluate the analgesic effect of breastfeeding during heel puncture in full term healthy newborn.

**Methods:**

We studied 200 healthy full term newborns (100 cases and 100 controls), proposing the puncture to mothers during breastfeeding, and explaining to them all the advantages of this practice. Pain assessment was evaluated by DAN scale (Douleur Aigue Nouveau ne scale).

**Results:**

The difference in score of pain according to the DAN scale was significant in the two groups of patients (p = 0.000); the medium score was 5.15 for controls and 2.65 for cases (newborns sampled during breastfeeding).

**Conclusion:**

Our results confirmed the evidence of analgesic effect of breastfeeding during heel puncture. This procedure could easily be adopted routinely in maternity wards.

## Introduction

Scientific studies show that even very premature newborns may experience sensation of distress which could unfavourably influence many clinical and behavioural parameters of their present and future [1]. Pain control in newborns is so primary importance, also stressed by the American Academy of Paediatrics [2]. The purpose of our study was comparing the analgesic effects of sucking own mother milk, versus alternative chances like caressing and/or pacifier, during routine invasive procedures in full-term newborns. The most painful routine invasive procedures in full-term newborns include venous blood sample and capillary heel stick blood sampling [3]. The analgesic effect of oral glucose 24% solution [4], pacifiers [5] and skin-to-skin contact [6] have already been demonstrated. The use of sucrose and/or pacifier for analgesia may interfere with a correct beginning of breastfeeding [7], so it may be an interesting alternative to test the analgesic effect of breastfeeding during painful procedures. In a recent review by Shah PS et al [8] breastfeeding is associated with changes in heart rate, duration of cry, percentage of crying time and a decrease of measured pain. Breastfeeding instead, does not seem to be favourable, if compared with higher glucose concentrations, with regards for crying duration, PIPP score and DAN score [9-11]. This suggests that neonates undergoing painful procedures may be breastfed or given expressed breast milk to obtain analgesic effect. This special power of breast-sucking may be linked to relational factors (skin-to-skin contact, nearness to mother, entertainment) [12,13] and to specific components of human milk like sugar [14] and triptophane [15] a melatonine precursor that enhances in neonates the production of beta endorphins [16], or the endogenous opioids like galattorphins [17]. This practice may be useful for driving on breastfeeding by frequent sucking and using mother's breast for comfort.

## Methods

We enlisted 200 full term healthy neonates (100 cases and 100 controls). We suggested to all mothers of completely or partial breastfed neonates the execution of metabolic screening from heel puncture during breast sucking, explaining to mothers the advantages of this practice.

**Figure 1:**
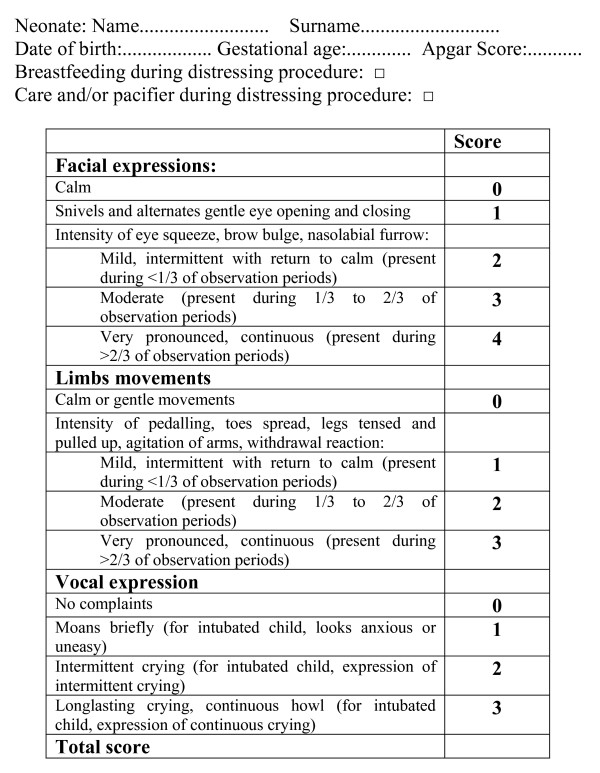
DAN scale.

The magnitude of pain in neonates was measured by DAN scale (Douleur Aigue Nouveau-nè scale – Figure 1), reckoning suffering in full-term healthy neonates by observing facial changes, limb movements and vocal expression [18]. This evaluation regarded both newborns undergoing capillary heel stick for screening while breastfed and control ones, i.e. children refusing breast during puncture, or formula fed ones, or babies who's mother had refused the procedure. In control cases, analgesia was provided by caressing and/or pacifier.

Every child was taken to his own mother and the puncture was performed after minimum two minutes of effective sucking. Capillary puncture was made on the postero-lateral area of the heel, using sterile click prick lancet (the kind of lancets we used were AMES MINILET LANCETS by Bayer). Every heel puncture was performed by dedicated nurses of Neonatology Division. Every nurse was trained about enlistment of neonates, about information of mothers, and especially about evaluation of pain according the standards of Dan Scale and attended a course of pain evaluation of newborn. For every enroled newborn, was filled a form including his/her name, date of birth, gestational age, Apgar score and way of punture (while breastfeeding or pacifier) (Figure 1).

A score from 0 (no pain) to 10 (highest pain) was attributed to each child, according to the intensity of pain [3,19]. After discharge, mothers were called to verify the outcome in breastfeeding at one month of life in the two groups. Scores obtained in the two groups were statistically analysed by χ2 test. Dates confirming homogeneity of groups were statistically compared with t-Student test. We used test for relating two proportions in feed evaluation of one month aged children.

We did not collect differences between caressing and pacifier in the control group.

Statistics data give signs of homogeneity of champions, in fact there are not elements influencing mothers in the choice of way of puncture, except for formula-fed neonates.

## Results

### Description of the studied population

The case group included 52 female neonates and 48 males tested with heel puncture while breastfed. The control group included 41 female and 59 male neonates. In the case group, 67 were born by vaginal delivery, 28 by caesarean section and 5 with use of vacuum extractor. In the control group, 59 were born by vaginal delivery and 41 by caesarean section. The mean gestational age in the case group was 39.42 weeks (DS1.27 and CI95 = 38.16–40.69), in controls one was 39.35 weeks (DS 1.31 and CI95 = 38.04–40.67). The mean first and fifth minute Apgar score in case group was 8.78 (DS 0.69 and CI 95 = 8.09–9.47) and 9.75 (DS 0.52 and CI 95 = 9.23–10); in the control group they were 8.76 (DS 0.85 and CI 95 = 7.90–9.61) and 9.69 (DS 0.71 and CI 95 = 8.98–10).

The mean birth weight in the first group was 3335.2 g (DS 434.82 and CI 95 = 2900.38 – 3770.02), in the second one 3333.45 g (DS 443.89 and CI 95 = 2889.55–3777.34). In the first group, at discharge 93 neonates were being breastfed and 7 were receiving breast milk and formula; at 72 hours after-discharge check-up, only one child receiving mixed feeding began to receive only formula. In the control group at discharge 90 neonates were fed by breast and 4 neonates were fed with human milk and formula. At the age of one month, in the examined group 65 neonates were exclusively breast fed, 14 were mainly breastfed, 13 partially breastfed, 8 were exclusively fed with formula. In controls group instead, at one month 63 neonates were exclusively breastfed, 13 were mainly breastfed, 7 were partially breastfed, 17 were fully formula fed. There is no evident statistical significant difference between the two groups regarding this last specific parameter, even if in control group, a larger number of neonates left own mother's milk. Rooming-in during hospital stay, was practised by 47 pairs of mother-child in the first group, and by 37 pairs in the second one. The mother's age was concerned, the mean age in the cases group was 32.18 years (DS 5.37 and CI 95 = 26.81–37.55). For half (50%) of the women it was their first pregnancy. Eleven percent of women had previous abortions and 5% voluntary interruption of pregnancy. Between pluriparous mothers, 78% had previously breastfed. In the control group, the mean age of mothers was 31.36 years (DS 4.93 and CI95 = 26.43–36.29); 43% of women were primiparous, 19% had previous abortions and 4% had voluntary pregnancy interruption. In pluriparous women, 78.95% had previously breastfed.

### Score for pain perception in neonates (DAN scale)

**Figure 2:**
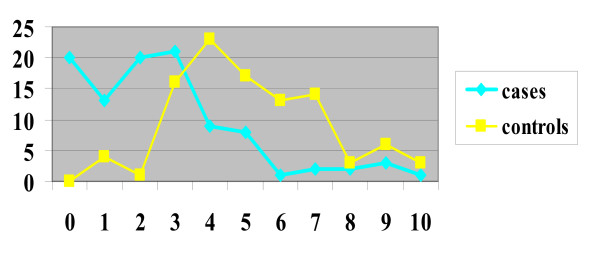
Graph comparing DAN scale total scores for cases and controls.

**Figure 3:**
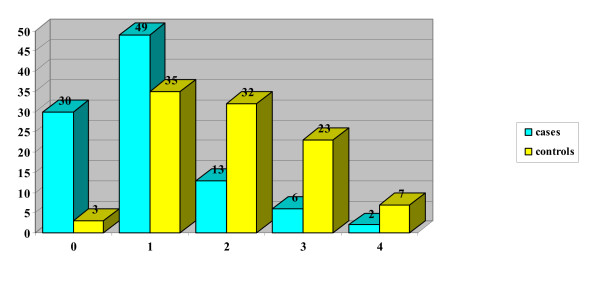
Facial expressions.

**Figure 4:**
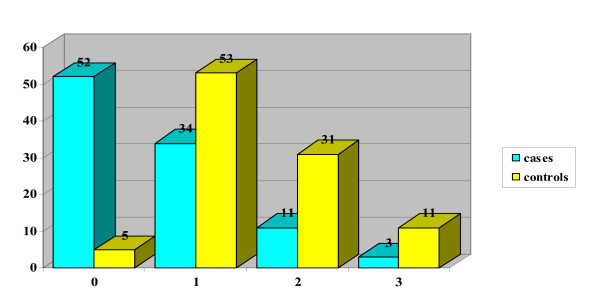
Limb movements.

**Figure 5:**
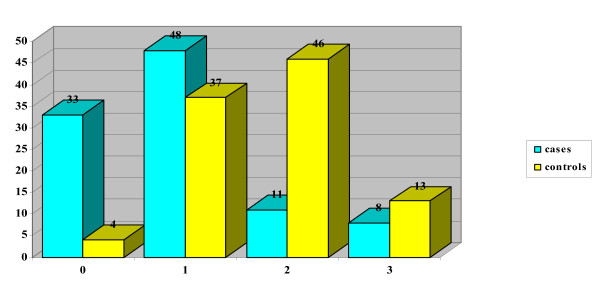
Vocal expression.

The mean DAN score in the case group (neonates with puncture during breastfeeding) was 2.65 (DS 2.31 and CI 95 = 0.34 – 4.96), for control group 5.15 (DS 2.07 and CI95 = 3.08–7.22). In the case group, 20 neonates obtained score 0, while no neonates in the control group got this score (Table 1 and Figure 2). From statistical analysis a significant difference resulted between the score obtained in the two groups (p = 0.000), even when the single parameters of the DAN scale were considered independently: face expression (p = 0.000) (Figure 3), limb movements (p = 0.000) (Figure 4), vocal expression (p = 0.000) (Figure 5).

**Table 1:** DAN scale total scores

	**Total**	**0**	**1**	**2**	**3**	**4**	**5**	**6**	**7**	**8**	**9**	**10**
cases	100	20	13	20	21	9	8	1	2	2	3	1

controls	100	0	4	1	16	23	17	13	14	3	6	3

## Discussion

Our results strengthen the already well-known analgesic effect of breastfeeding. This analgesic effect may be successfully exploited for minor distressing procedures in full term healthy neonates. The choice of this easy and effective method, is useful in reducing all external interferences with a beginning of breastfeeding, while other analgesic systems such as pacifiers or sucrose might disrupt a good start at breastfeeding. Moreover, stressing the curative effects of human milk, may be an important confidence boost for mothers, hopefully rendering breastfeeding easier to pursue (regarding this point, we need a longer follow-up). In our study, doing a heel lance without sucking, does not seem to influence the kind of feeding at one month of age. In our next collection of data, we would include a further group of newborns – the ones pacified with sucrose.

In our opinion, this analgesic system may easily become a routine way of performing heel lance in maternity wards. This procedure may be suggested to available mothers of newborns who are sucking effectively, and possibly also extended to other painful minor procedures like intramuscular injection or venous puncture. Further studies might consider this method in older children, for instance during vaccination.

## Competing interests

The authors declare that they have no competing interests.

## Authors' contributions

EU and SP conceived of the study and participated in its design and coordination. AP, VA, GA, MT, AMZ, MBR, DZ, CT and MA participated in the collection of data. MV and LG carried out the analyses. MC participated in the design of the study and performed the statistical analysis. All authors read and approved the final manuscript.
